# Experimental Study on the Dilatancy and Energy Evolution Behaviors of Red-Bed Rocks under Unloading Conditions

**DOI:** 10.3390/ma16175759

**Published:** 2023-08-23

**Authors:** Zhao-Qiang Zheng, Huai-Zhong Liu, Li Zhuo, Ming-Li Xiao, Hong-Qiang Xie, Jiang-Da He, Ming-Liang Peng

**Affiliations:** 1 State Key Laboratory of Hydraulics and Mountain River Engineering, College of Water Resource and Hydropower, Sichuan University, Chengdu 610065, China; zhengzhaoqiang@stu.scu.edu.cn (Z.-Q.Z.);; 2 Sichuan Water Development Investigation, Design & Research Co., Ltd., Chengdu 610072, China

**Keywords:** red-bed rock, dilatancy angle, dissipated energy, plastic strain, laboratory test

## Abstract

Surrounding rock deformation and consequent support failure are the most prominent issues in red-bed rock tunnel engineering and are mainly caused by the effects of unloading, rheology, and swelling. This study investigated the mechanical responses of two kinds of red-bed mudstone and sandstone under unloading conditions via laboratory observation. Volume dilation was observed on the rocks during unloading, and the dilatancy stress was linear with the initial confining pressure. However, the ratios of dilatancy stress to peak stress of the two rocks kept at a range from 0.8 to 0.9, regardless of confining pressures. Both the elastic strain energy and the dissipated energy evolved synchronously with the stress–strain curve and exhibited conspicuous confining pressure dependence. Special attention was paid to the evolution behavior of the dilatancy angle. The dilatancy angle changed linearly during unloading. When the confining pressure was 10 MPa, the dilatancy angle of mudstone decreased from 26.8° to 12.5° whereas the dilatancy angle of sandstone increased from 34.6° to 51.1°; when the confining pressure rose to 25 MPa, the dilatancy angle of mudstone and sandstone decreased from 45.8° to 17.4° and increased from 21.7° to 39.5°, respectively. To further understand the evolution of the dilatancy angle, we discussed the links between the variable dilatancy angle and the processes of rock deformation and energy dissipation.

## 1. Introduction

**Figure 1:**
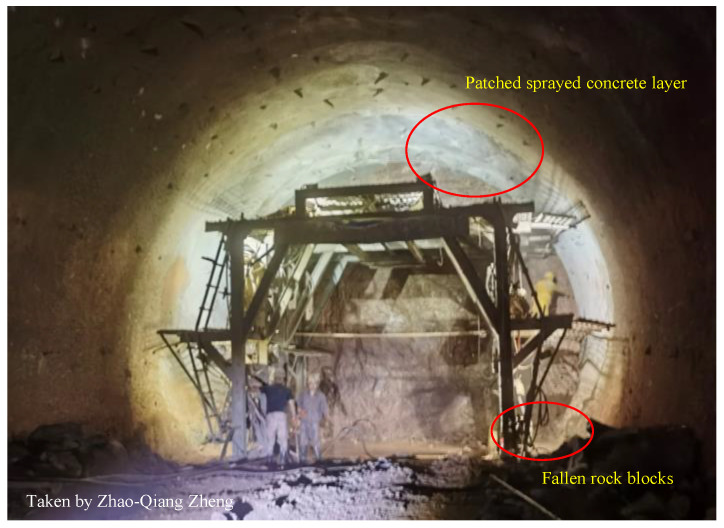
Excavation-induced rock deformation and consequent support structure damage in a tunnel project constructed in red-bed strata.

Red-bed rocks, mainly composed of mudstone and sandstone, are characterized by weak cementation and consequent low strength. In underground engineering practice, red-bed rocks usually exhibit large deformation and cause support structure damage. For example, a water conveyance tunnel constructed in interlayered mudstone–sandstone strata suffered continuous concrete spraying layer cracking and block falling in the top arch part after excavation, seriously affecting the construction safety and process of the project, as shown in Figure 1.

The excavation-induced unloading effect is considered a major reason for the large deformation phenomenon of the surrounding rock [1], and researchers have conducted extensive studies on this issue [2,3]. Volume dilatancy phenomena have been widely observed in laboratory studies and attracted strong interest from researchers [4,5,6,7]. The dilatancy boundary stress, also named yield stress, usually exhibits positive linear or nonlinear relationships with the initial confining pressure [8,9,10], and the plastic volumetric strain accumulates acceleratively with decreasing confining pressure [11,12,13]. As the determination of dilatancy angle needs accurate strain measurement, researchers made efforts in improving the strain measuring approaches at the laboratory scale [14,15].

Since Reynolds first proposed the concept of dilatancy angle for granular materials [16], it has been well studied and widely adopted to describe the volume dilatancy behaviors of solid materials [17,18]. Vermeer and Brost [19] systematically illustrated the mechanical meaning of the dilatancy angle and investigated its variation characteristics via theoretical and experimental analyses. Salehnia et al. [20] analyzed the influence of dilatancy angle variation on the deformation of the surrounding rock mass of tunnels. Alejano, Walton, and their colleagues [10,21,22,23,24] conducted comprehensive studies on rock materials’ dilatancy angle and found it is highly dependent on the confining pressure and deformation history. Molladavoodi and Rahmati [25] studied the influence of dilation angle variation on the deformation of the plastic zone around tunnels through analytical and numerical methods. In the studies conducted by Zhang et al. [26] and Wang et al. [27], the dilatancy angle gets larger under higher confining pressure, but Zhao and Li [28] and Chen et al. [29] observed the opposite relationships where the dilatancy angle decreased as the confining pressure rose. Among the research on the plastic deformation dependency of dilatancy angle, the plastic shear strain was found to be a suitable internal parameter to quantitatively characterize the evolution of the dilatancy angle [20]. Zhao et al. [30] and Tsegaye et al. [31]. found an attenuative increasing–decreasing relationship between the dilatancy angle and plastic shear strain. However, some different results were observed by other investigators. Wang et al. [32] found on three sedimentary rocks that the dilatancy angle monotonically decreased with increasing plastic shear strain under the uniaxial compression condition. Dai et al. [33] conducted a series of triaxial unloading tests on granite and found the dilatancy angle evolved in different manners when the confining pressure was unloaded at different rates. In conclusion, the evolution of the dilatancy angle depends on not only the intrinsic properties of the material but also external factors.

According to the law of energy conservation, the deformation and failure of rocks are essentially driven by energy conversion. Energy evolution characteristics of different rocks under unloading conditions have been comprehensively studied [34,35]. Huang and Li [36] investigated the conversion of strain energy of marble under increasing axial stress and decreasing confining pressure. They found the change in elastic strain energy closely following the axial stress–strain curves and the dissipated energy increasing monotonically and acceleratively during loading. Similar phenomena were observed by other researchers on marbles [11,37], granites [38,39], dacites [40], and hard sandstones [41,42,43]. However, differences exist in the dissipated energy between different rocks. Liu et al. [44] found the dissipated energy ratio of a sandstone grew acceleratively with increasing axial stress. However, the dissipated energy ratio of another sandstone reported by Li et al. [45] showed rather complicated evolution behaviors with varying moisture content. Moreover, the dissipation behaviors of strain energy show a strong dependence on the unloading rate [46]. Fedotova et al. [47] established the connection between energy accumulation capability and the strength and deformation characteristics of hard rocks. Yet, to the best of the authors’ knowledge, the mechanical responses of red-bed rock under triaxial unloading paths have rarely been studied from the perspective of volume dilation and energy conversion. Nor has the possible correlation between the evolution behaviors of the dilatancy angle and dissipated energy been comprehensively investigated.

In this study, we investigated the mechanical properties of sandstone and mudstone collected from red-bed strata under unloading conditions by means of laboratory experiments. The elastic and plastic deformation characteristics were analyzed. Attention was paid to the volume dilatancy behavior and the energy conversion process. The evolution trends of the dilatancy angle were carefully analyzed. Further, we investigated the correlations between the dilatancy angle evolution and the rocks’ failure process and found the possible relevance between the dilatancy angle evolution and energy dissipation. The findings can enrich the knowledge of the deformation behaviors of red-bed rock and provide scientific support for engineering practice.

## 2. Materials and Methods

### 2.1. Specimen Preparation

**Figure 2:**
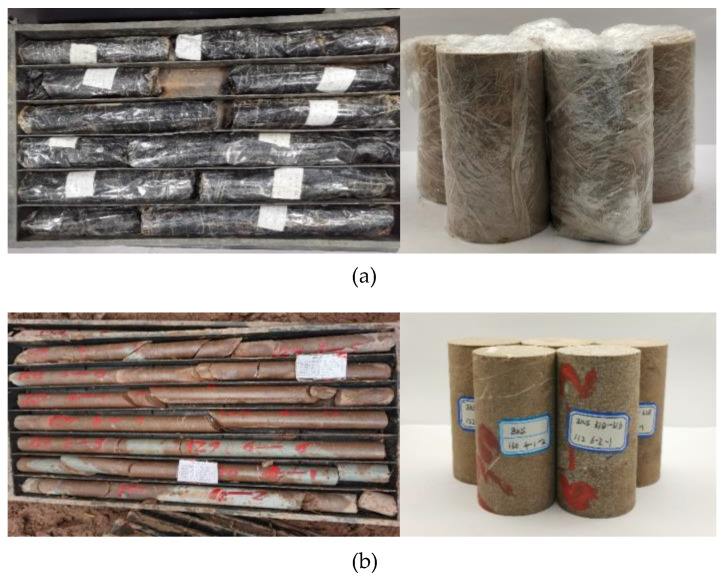
Drill cores and processed specimens, (**a**) mudstone and (**b**) sandstone.

The studied rocks are collected from a tunnel project in a Lower Jurassic interlayered mudstone–sandstone stratum in Southwest China. The cores were wrapped and then processed into cylinders 100 mm in height and 50 mm in diameter, as shown in Figure 2. The basic physical parameters of the mudstone and the sandstone are presented in Table 1. Despite similar natural densities, the porosities and P-wave velocities of the two rocks are quite different. X-ray diffraction and X-ray fluorescence analyses revealed the mineral components of the rock. As shown in Table 2, the main diagenetic minerals are quartz and feldspar, calcareous minerals take 20% to 30%, and the clayey minerals are mainly chlorite and kaolinite. Note that kaolinite takes 10.8% of the mass of the mudstone, but it is hardly detected in the sandstone.

### 2.2. Experimental Equipment and Test Scheme

**Figure 3:**
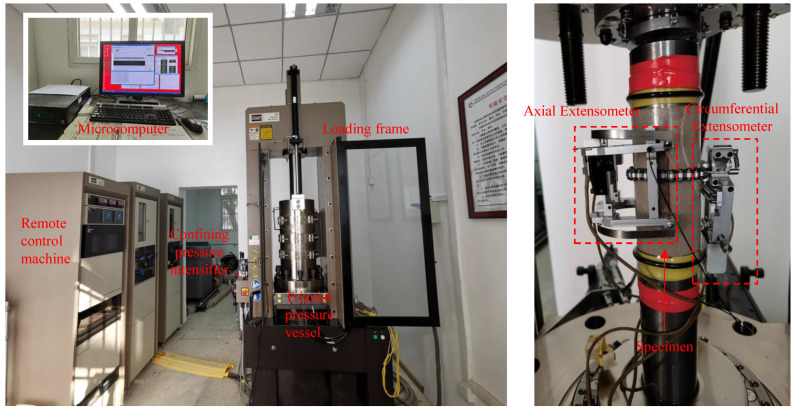
Illustration of the testing equipment.

The laboratory tests were conducted on the MTS 815 Rock and Concrete Mechanics Testing System of Institute of Mountain Hazards and Environment, Chinese Academy of Science. It allows a maximum axial load of 1000 kN and a maximum confining pressure of 80 MPa. The specimen was wrapped by a thin Teflon jacket and PVC tape to prevent oil. The axial and lateral deformation of specimens can be measured by the axial and circumferential extensometer, respectively, as shown in Figure 3.

**Figure 4:**
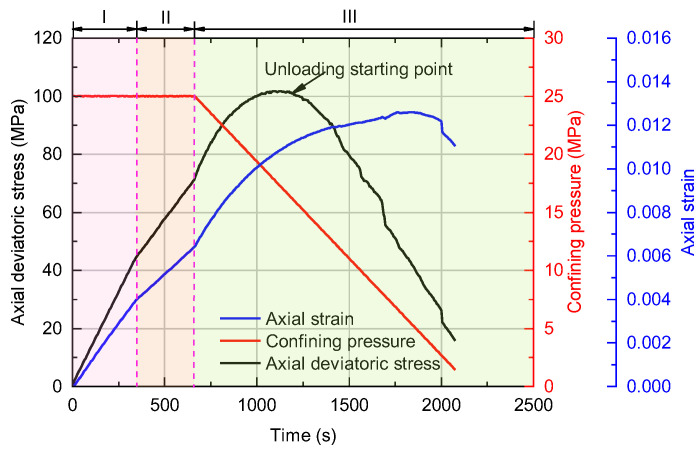
Typical loading path of a triaxial unloading confining pressure (TUCP) test, Phase I axial force control mode, Phase II axial deformation control mode, and Phase III confining pressure unloaded.

Both conventional triaxial compression (CTC) tests and triaxial unloading confining pressure (TUCP) tests were conducted on the specimens under the confining pressures of 10 MPa, 20 MPa, and 25 MPa, respectively. In conventional triaxial compression tests, we first lift the confining pressure to the preset value at a rate of 0.1 MPa/s, then keep the axial deformation rate at 0.06 mm/min throughout the loading process until the specimen fails. A representative loading path of TUCP tests is presented in Figure 4. First, the confining pressure was loaded to the preset value at a rate of 0.25 MPa/s. Then, axial loading was conducted at a rate of 10 kN/min to about 60% of the corresponding mean peak force. After that, the axial loading mode switched to deformation-controlled mode with an axial deformation rate of 0.06 mm/min. When the axial force reached 70% of the mean peak force in CTC tests, we continued to increase the axial deviatoric stress at a circumferential deformation rate of 0.06 mm/min while unloading the confining pressure at a rate of 1 MPa/min until the specimen failed. In this study, a constant unloading rate of 0.05 MPa/s was selected. As suggested by Wang et al. [48], red-bed rock exhibited lower strengths at higher unloading rates within 0.05 MPa/s to 0.2 MPa/s, but the failure mode of rocks hardly changed. As the main object of the study is to comprehend the mechanical behaviors of red-bed rock under general unloading conditions but not to investigate the unloading rate effect of the rocks, a uniform unloading rate was adopted.

## 3. Results

### 3.1. Stress–Strain Relationship

**Figure 5:**
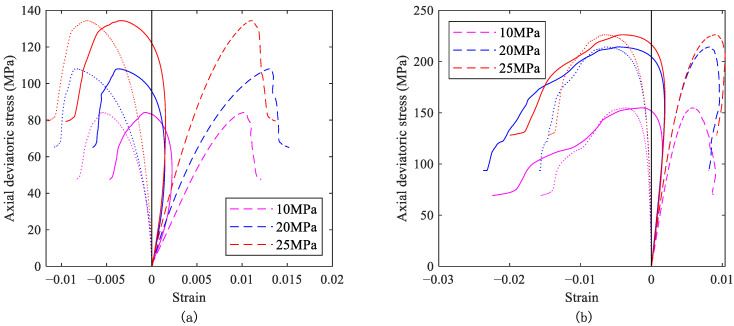
Typical stress–strain curves in conventional triaxial compression (CTC) tests conducted on (**a**) the mudstone and (**b**) the sandstone. Dashed lines, chain lines, and solid lines represent the axial, lateral, and volumetric stress–strain curves, respectively.

**Figure 6:**
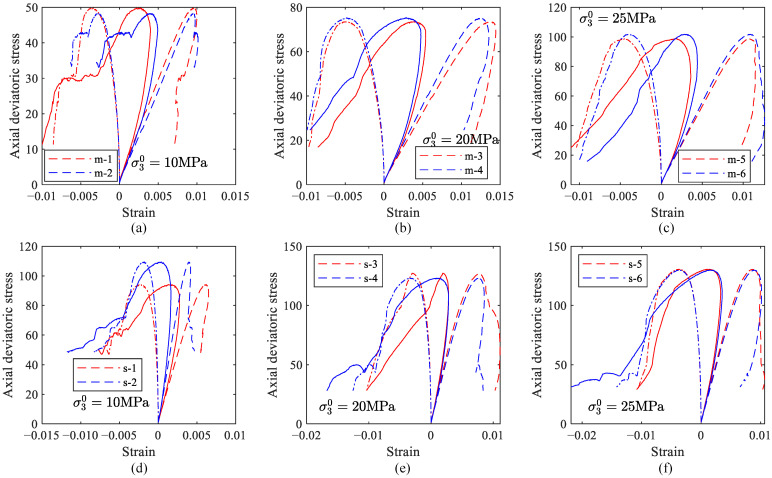
Typical stress–strain curves in triaxial unloading tests, (**a**–**c**) mudstone and (**d**–**f**) sandstone, $\sigma_{3}^{0}$ refers to the initial confining pressure during the test. Dashed lines, chain lines, and solid lines represent the axial, lateral, and volumetric stress–strain curves, respectively.

Figure 5 shows the stress–strain curves of the representative specimens in CTC tests, where positive strains represent compression deformation. Under confining pressures, the pre-peak stress–strain curves demonstrate elastic–plastic characteristics and the void compaction phase is absent due to initial hydrostatic compaction. During the post-peak stage, stress–strain curves experience a steep descent and then enter the residual deforming phase. As $\sigma_{3}$ increases, the peak strengths and the peak axial strains of the rocks increase. In Figure 5a, the peak axial strains of the mudstone seem to be larger than the peak lateral strains. In comparison, the sandstone demonstrates significant post-peak dilatancy characteristics, as shown in Figure 5b. Moreover, under the same confining pressure, the peak axial strain of the sandstone is smaller than that of the mudstone, indicating the higher brittleness of the sandstone. Figure 6 demonstrates the stress–strain curves of the rocks in TUCP tests. Differences exist in the stress–strain curves between the CTC and TUCP tests. The peak volumetric strains are negative (compressive) in TUCP tests but positive (dilatant) in CTC tests. Moreover, the post-peak axial stress–strain curves in CTC tests expand with decreasing axial stress, but those in TUCP tests evolve in the opposite way.

**Figure 7:**
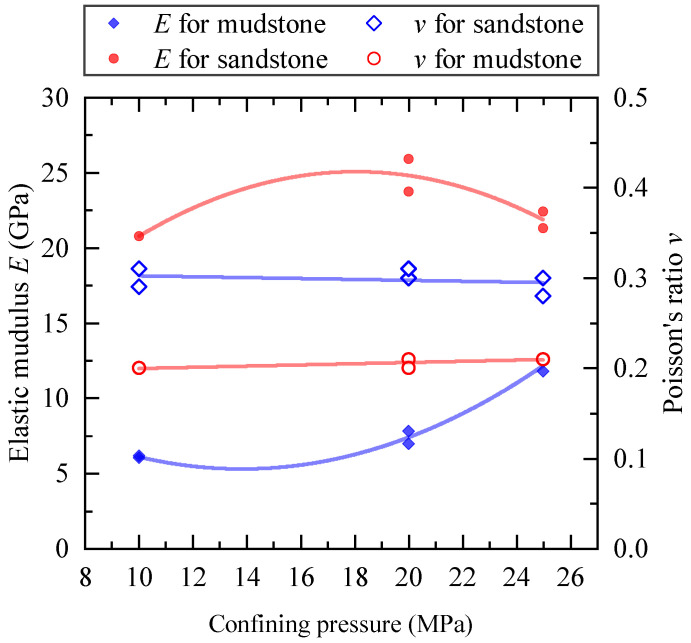
Elastic parameters of the rocks in TUCP tests.

The elastic modulus E and the Poisson’s ratios ν of the specimens in TUCP tests are calculated by Equations (1) and (2), and the results are presented in Table 3. Generally, mudstone has larger Poisson’s ratios but lower elastic moduli in contrast to sandstone. By plotting the elastic parameters against the initial confining pressure, we can find that the Poisson’s ratios of both rocks hardly change with the varying confining pressure, but the elastic moduli change nonlinearly with the initial confining pressure, as shown in Figure 7. The decrease in the elastic moduli from $\sigma_{3}^{0}=20\;\mathrm{MPa}$ to $\sigma_{3}^{0}=25\;\mathrm{MPa}$ may be caused by the discreteness of the specimens.

(1) $$\nu =\frac{△\varepsilon_{1}△\sigma_{3}-△\varepsilon_{3}△\sigma_{1}}{△\varepsilon_{1}△\sigma_{3}+△\varepsilon_{1}△\sigma_{1}-2△\varepsilon_{3}△\sigma_{3}}$$

(2) $$E=\frac{△\sigma_{1}}{△\varepsilon_{1}}-2\nu \frac{△\sigma_{3}}{△\varepsilon_{1}}$$

where △ represents the increment of a variable.

**Figure 8:**
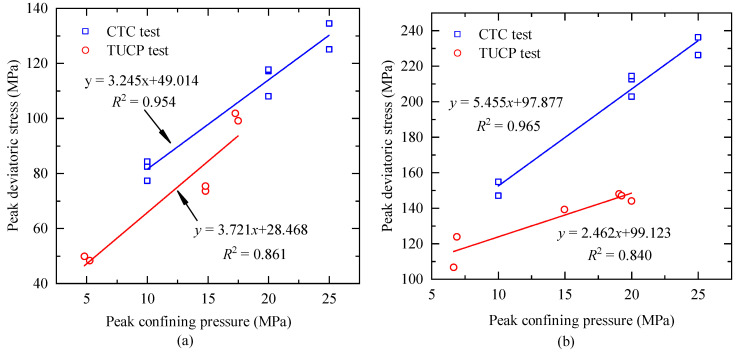
The relationships between the peak deviatoric stress and the peak confining pressure of (**a**) mudstone and (**b**) sandstone.

Figure 8 depicts the relationships between the peak deviatoric stress $\sigma_{\mathrm{dev}}^{p}$ and the peak confining pressure $\sigma_{3}^{p}$ in CTC and TUCP tests. As shown by Figure 8a, in both CTC and TUCP tests, a linear relationship exists between the peak deviatoric stress and the confining pressure of the mudstone. Similar relationships can also be observed on the sandstone from Figure 8b. For both rocks, the peak deviatoric stresses in TUCP tests are lower than those in CTC tests under the same peak confining pressure, but the difference seems larger for the sandstone, which indicates the sandstone suffers more severe damage during unloading. Then, according to Equation (3), the Mohr-Coulomb shearing strength parameters of the rocks were calculated, and the results are listed in Table 3. Compared to the strength parameters in CTC tests, the cohesion strength of the mudstone decreases by 45% under the unloading path, and the friction angle gains a moderate increase of 6%. However, the strength of the sandstone demonstrates significant susceptibility to the unloading effect, where the cohesion strength increases by 38% under the unloading condition, and the friction angle decreases by 40%.

(3) $$\sigma_{\mathrm{dev}}=\sigma_{1}-\sigma_{3}=\frac{2\sin\varphi }{1-\sin\varphi }\sigma_{3}+\frac{2c\cos\varphi }{1-\sin\varphi }$$

**Figure 9:**
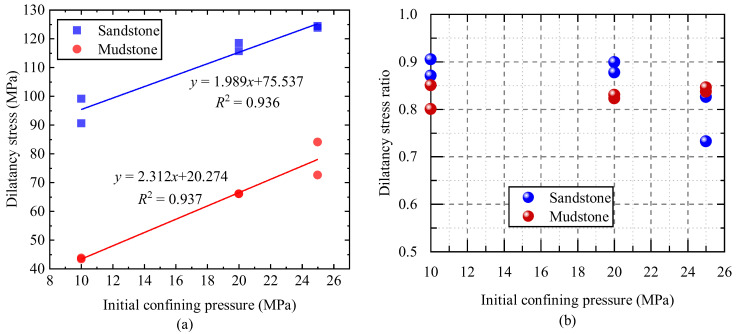
Dilatancy stress thresholds of rocks in TUCP tests under different initial confining pressures, (**a**) dilatancy stresses, and (**b**) ratios of dilatancy stress to peak deviatoric stress.

Dilatancy stress $\sigma_{1}^{d}$ refers to the axial deviatoric stress from which the volumetric strain converts from compression to dilation. Table 3 also presents the dilatancy stresses of the rocks in TUCP tests. For both rocks, the dilatancy stress increases linearly with the initial confining pressure, as shown in Figure 9a. By normalizing the dilatancy stress with the corresponding $\sigma_{1}^{p}$, Figure 9b illustrates the relationships between the dilatancy stress ratio and the initial confining pressure. As can be seen, the dilatancy stress ratios of both rocks change slightly from 0.8 to 0.9 with the varying initial confining pressure. Thus, the dilatancy stress ratio of the studied red-bed rock can be roughly considered independent of the initial confining pressure.

### 3.2. Plastic Deformation and Dilatancy Behaviors

As can be seen in Figure 6, all the stress–strain curves during the deviatoric stress loading stage are convex, and this deformation process is always accompanied by nonlinear plastic deformation. In this section, the plastic deformation of the rocks is analyzed.

Assuming that the elastic parameters do not degrade during loading, in the principal stress–strain coordinate system, the plastic strain components can be expressed by Equation (4).

(4) $$\varepsilon_{i}^{p}=\varepsilon_{i}-\left[\frac{\sigma_{i}}{E}-\left(1-\delta_{ij}\right)\nu \frac{\sigma_{j}}{E}\right]$$

where $\delta_{ij}$ is the Kronecker symbol.

**Figure 10:**
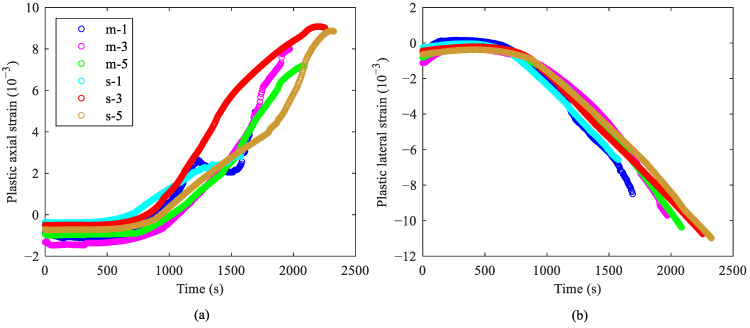
Plastic strains of the rocks in TUCP tests in (**a**) axial direction and (**b**) lateral direction.

Then, we calculated the axial and plastic lateral strains during TUCP tests and presented the results in Figure 10. In Figure 10a, the plastic axial strain goes through generally two periods. In the first phase, the plastic axial strain is negative and merely increases, which means the axial deformation is mainly elastic. In the second phase, the plastic axial strain starts to accumulate. Correspondingly, the plastic lateral strain first keeps invariable or grows negatively for a while and then increases linearly, as shown in Figure 10b. In some published research [11,12], the plastic strains develop unstably during loading and show significant confinement dependence. However, for the studied mudstone and sandstone, the evolutions of the plastic strains seem to follow similar trends during loading under different initial confinement, and the plastic strain rates almost keep invariable in the second phase until failure.

In addition, two parameters, the plastic shear strain γ [49] and the plastic volumetric strain $\varepsilon_{v}^{p}$, are usually used to describe the plastic deformation of geomaterials, and they can be calculated according to Equations (5) and (6).

(5) $$\gamma^{p}=\varepsilon_{1}^{p}-\varepsilon_{3}^{p}$$

(6) $$\varepsilon_{v}^{p}=\varepsilon_{1}^{p}+2\varepsilon_{3}^{p}$$

**Figure 11:**
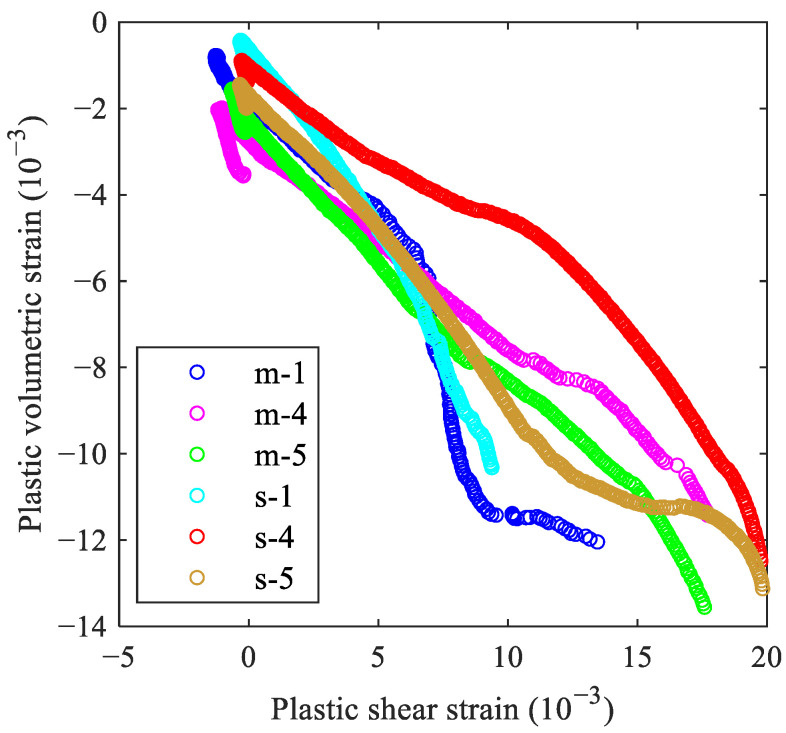
Plastic volumetric strain evolution versus plastic shear strain in TUCP tests.

As shown in Figure 11, the relationships between $\varepsilon_{v}^{p}$ and γ in TUCP tests on both rocks share similar characteristics under different confinements. At the beginning of loading, the plastic shear strain and the plastic volumetric strain grow negatively, which means the specimen is compressed during this period. Later, the plastic shear strain develops positively, while the plastic volumetric strain converts from compressive to dilative. Next, the plastic volumetric strain evolves linearly and finally nonlinearly with the increasing plastic shear strain, indicating the non-controllable failure process of the specimen during the post-peak loading period. No pronounced difference is observed between different rocks, and the relationship between the volumetric and plastic shear strain seems to be independent of the confining pressure.

To further understand the evolution behaviors of the plastic strains, a new parameter $\kappa_{i}(i=1,3)$ is defined to estimate the behaviors of plastic strain evolution. $\kappa_{i}$ is precisely defined with Equation (7) to describe the change in the plastic deformation behaviors before and after the dilatancy stress

(7) $$\kappa_{i}=\frac{\varepsilon_{i}^{p,p}-\varepsilon_{i}^{p,d}}{\varepsilon_{i}^{p,p}-\varepsilon_{i}^{p,i}}(i=1,3),$$

where variables $\varepsilon_{i}^{p,p}$, $\varepsilon_{i}^{p,d}$, and $\varepsilon_{i}^{p,i}$ are the plastic strain component in a certain direction at the initial loading, dilatancy stress, and peak stress points, respectively. $\kappa_{i}$, thus, refers to the relationships between the plastic strain increments accumulated from the dilatancy point to the peak point and the total plastic strain increments during pre-peak loading, and a large $\kappa_{i}$ means more plastic strain generates after the dilatancy stress point.

**Figure 12:**
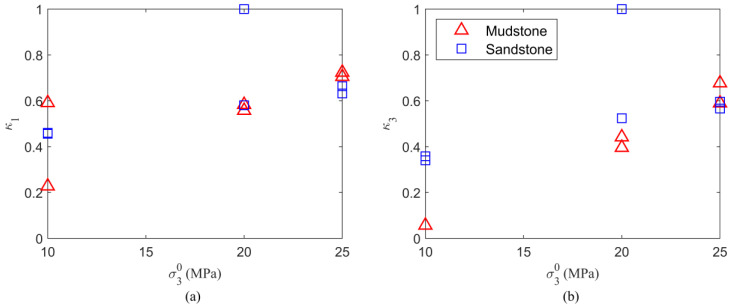
Ratios of axial and plastic lateral strain increment at dilatancy points versus initial confining pressures in TUCP tests of (**a**) the mudstone and (**b**) the sandstone.

The values of $\kappa_{i}$ were plotted against the initial confining pressure $\sigma_{3}^{0}$ as shown in Figure 12. For both the axial and lateral directions, $\kappa_{i}$ increases linearly with $\sigma_{3}^{0}$, which means more plastic strains generate during the post-dilatancy stress phase under higher initial confining pressure. It is caused by the increase in the dilatancy stress with rising confining pressure. Figure 9 depicts the relationship between the dilatancy stress $\sigma_{1}^{d}$ and the initial confining pressure $\sigma_{3}^{0}$. As seen in Figure 9a, the dilatancy stress $\sigma_{1}^{d}$ of the mudstone increases linearly as the initial confining pressure $\sigma_{3}^{0}$ rises. Thus, higher deviatoric stresses promote the growth of plastic strains, and $\kappa_{i}$ consequently increases.

With the development of the plastic volumetric strain, the axial stress–volumetric strain curves convert from compaction to dilation. The dilatancy angle ψ is generally used in describing the dilatancy behavior of a material, and it can be calculated based on Equation (8) [50],

(8) $$\sin\psi =\frac{{\dot{\varepsilon }}_{v}^{p}}{{\dot{\varepsilon }}_{v}^{p}-2{\dot{\varepsilon }}_{1}^{p}}$$

where ${\dot{\varepsilon }}_{v}^{p}$ and ${\dot{\varepsilon }}_{1}^{p}$ are the volumetric and plastic axial strain rate, respectively.

**Figure 13:**
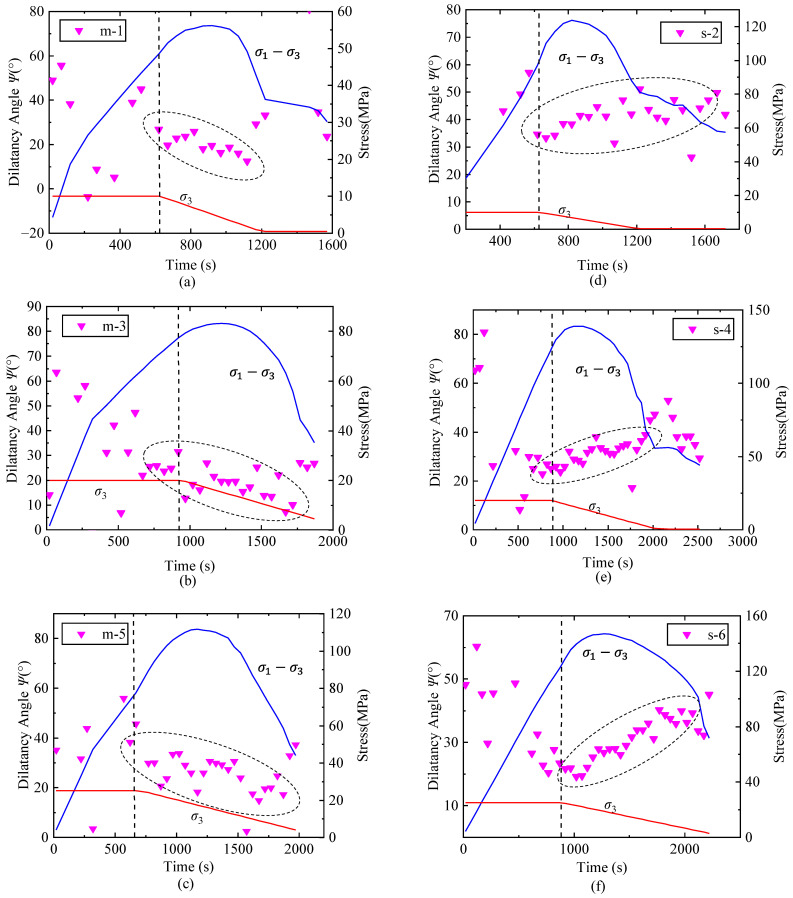
Evolution of dilatancy angle ψ in TUCP tests, (**a**) m-1 with $\sigma_{3}^{0}=10\;\mathrm{MPa}$, (**b**) m-3 with $\sigma_{3}^{0}=20\;\mathrm{MPa}$, (**c**) m-5 with $\sigma_{3}^{0}=25\;\mathrm{MPa}$, (**d**) s-2 with $\sigma_{3}^{0}=10\;\mathrm{MPa}$, (**e**) s-4 with $\sigma_{3}^{0}=20\;\mathrm{MPa}$, and (**f**) s-6 with $\sigma_{3}^{0}=25\;\mathrm{MPa}$, where $\sigma_{3}^{0}$ is the initial confining pressure. The vertical dashed black line indicates the onset of confining pressure unloading.

We calculated the dilatancy angles of all the specimens throughout the loading phases in TUCP tests, and some representative results are presented in Figure 13. Along with the dilatancy angles, the load paths are also demonstrated. Figure 13a–c show the dilatancy angles of the mudstone under initial confining pressures of 10 MPa, 20 MPa, and 25 MPa, respectively. The dilatancy angles distribute randomly during the phase where the confining pressure is kept constant. Then, the dilatancy angle experiences a mild decrease during the unloading phase. When $\sigma_{3}^{0}=10\;\mathrm{MPa}$, the dilatancy angles decrease from 26.8° to 12.5°; when $\sigma_{3}^{0}=20\;\mathrm{MPa}$, the dilatancy angles decrease from 31.5° to 10.1°; when $\sigma_{3}^{0}=25\;\mathrm{MPa}$, the dilatancy angles decrease from 45.8° to 17.4°. And a sudden increase appears in the dilatancy angle when the specimen approaches failure.

Figure 13d–f demonstrate the dilatancy angle of the sandstone in TUCP tests under different initial confining pressures. Different from the mudstone, the dilatancy angle of the sandstone keeps rising during the confining pressure unloading period. When $\sigma_{3}^{0}=10\;\mathrm{MPa}$, the dilatancy angles increase from 34.6° to 51.1°; When $\sigma_{3}^{0}=20\;\mathrm{MPa}$, the dilatancy angles increase from 26.9° to 47.3°; when $\sigma_{3}^{0}=25\;\mathrm{MPa}$, the dilatancy angles increase from 21.7° to 39.5°.

### 3.3. Energy Conversion under Unloading Conditions

In essence, the deformation of geomaterials under loads is driven by energy conversion, and analyzing the energy conversion process can help understand the mechanisms of rock’s mechanical behaviors. For the sake of simplicity, three assumptions are adopted in this study: the rock materials are homogenous, the stress field in the specimen is uniform, and the kinetic and thermal energy dissipation are ignored. Thus, the input energy converts into two parts during loading: the elastic strain energy stored in the material and the dissipated energy consumed by micro-fracturing and plastic deformation, and the energy conversion behaviors of the specimen can be represented by that of a microbody. Let the volume of the microbody equal 1, the elastic strain energy density $U^{e}$ can be calculated by Equation (9), and the total input energy density U can be calculated by Equation (10). Then, the dissipated energy density $U^{d}$ is determined by Equation (11).

(9) $$U^{e}=\frac{1}{2E}\left[\sigma_{1}^{2}+2\sigma_{3}^{2}-2\mu \left(2\sigma_{1}\sigma_{3}+\sigma_{3}^{2}\right)\right]$$

(10) $$U=\int \sigma_{i}d\varepsilon_{i}=\int_{0}^{\varepsilon_{1}}\sigma_{1}d\varepsilon_{1}+2\int_{0}^{\varepsilon_{3}}\sigma_{3}d\varepsilon_{3}$$

(11) $$U^{d}=U-U^{e}$$

According to Equations (9)–(11), the energy state of the specimen during loading can be determined. However, the input work during initial hydrostatic loading cannot be determined due to the lack of stress–strain data records. Thus, when analyzing the energy conversion behaviors during the deviatoric loading process, the elastic strain energy stored during initial hydrostatic loading should be excluded from the current elastic strain energy, and the excluded part can be expressed as Equation (12) [51].

(12) $$U_{0}^{e}=\frac{3\left(1-2\nu \right)}{2E}{\left(\sigma_{3}^{0}\right)}^{2}$$

**Figure 14:**
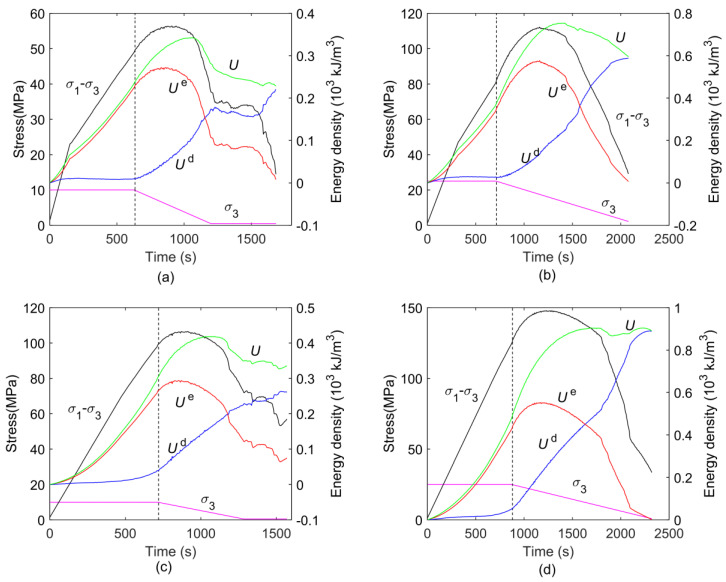
Energy evolution during TUCP tests, (**a**) m-1 with $\sigma_{3}^{0}=10\;\mathrm{MPa}$, (**b**) m-5 with $\sigma_{3}^{0}=25\;\mathrm{MPa}$, (**c**) s-1 with $\sigma_{3}^{0}=10\;\mathrm{MPa}$, and (**d**) s-5 with $\sigma_{3}^{0}=25\;\mathrm{MPa}$, where $\sigma_{3}^{0}$ is the initial confining pressure.

Figure 14a,b show the energy evolution behaviors of specimens m-1 and m-5, respectively. During the earlier loading phase with invariable $\sigma_{3}$, the elastic strain energy increases as the deviatoric stress rises, and the dissipated energy accumulates slowly, which indicates the deformation during this period is of elastic nature. As confining pressure unloading starts, the restored elastic strain gradually releases, and released energy is acceleratively dissipated in the forms of plastic work and fracture work or partially converts into surface energy. In the post-peak phase, energy is continuously dissipated with the developing irreversible deformation, and less elastic strain energy is held in the specimen. In Figure 14c,d, the sandstone specimens demonstrate a similar energy evolution trend to the mudstone. But subtle difference exists between the rocks. As can be seen in Figure 14c,d, the dissipated energy slightly increases during the period with invariable confining pressure. Then, from the onset of unloading, the dissipated energy starts to accumulate at a constant rate until the axial stress–strain curve meets the first post-peak drop. When the specimen fails, almost all the restored elastic strain energy is consumed by irreversible deformation. Overall, the elastic strain energy evolves closely following the stress–strain behavior, and the maximum input energy during loading is dependent of the initial confinement. Under the lower initial confining pressure of 10 MPa, as shown in Figure 14a,c, the maximum input energy densities of the mudstone and the sandstone are less than 0.4 × 10^3^ kJ/m^3^ and 0.5 × 10^3^ kJ/m^3^, respectively. However, when $\sigma_{3}^{0}$ rises to 25 MPa, the maximum input energy density values of the mudstone and the sandstone are close to 0.8 × 10^3^ kJ/m^3^ and 1.0 × 10^3^ kJ/m^3^, respectively.

**Figure 15:**
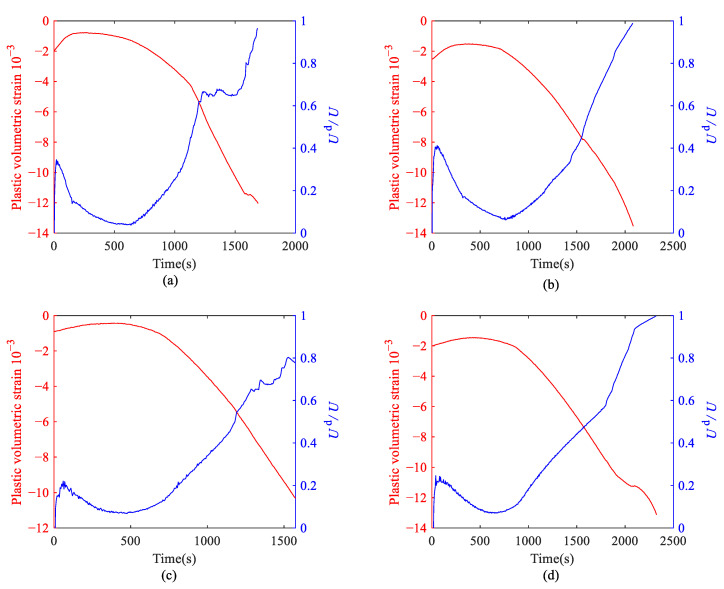
Evolution of plastic volumetric strain and ratios of cumulated dissipated energy density to the total input energy density during TUCP tests, (**a**) m-1 with $\sigma_{3}^{0}=10\;\mathrm{MPa}$, (**b**) m-5 with $\sigma_{3}^{0}=25\;\mathrm{MPa}$, (**c**) s-1 with $\sigma_{3}^{0}=10\;\mathrm{MPa}$, and (**d**) s-5 with $\sigma_{3}^{0}=25\;\mathrm{MPa}$, where $\sigma_{3}^{0}$ is the initial confining pressure.

Further, we analyzed the changes in the dissipated energy ratio during the loading process. As energy is consumed by micro-fracturing and plastic deformation, and these mechanical phenomena usually cause irreversible volume change in the material, we also investigated the plastic volumetric strain change throughout the loading duration. The dissipated energy ratio $U^{d}/U$ and the plastic volumetric strain are plotted against the elapsed loading time in Figure 15. For both the mudstone and sandstone, the plastic volumetric strain evolves negatively during the early loading stage, which means the specimen is mainly compressed. Meanwhile, a sharp increase in $U^{d}/U$ can be observed at the very beginning and then reduces moderately at an attenuative rate. During this period, energy is consumed mainly by initial void closure, and most input energy converts into elastic strain energy. As a result, the value of $U^{d}/U$ is relatively low and keeps decreasing as initial void closure gradually completes. Then, the irreversible strain developed with the increasing stress due to microscopic crack propagation. Since the microscopic cracks were tensile, the specimen exhibited volume dilation; thus, the plastic volumetric strain developed positively. Meanwhile, more energy was consumed by crack expansion, and the ratio of dissipated energy gradually increased. When the specimen approached failure, cracks expanded violently; as a result, the volumetric plastic strain developed rapidly and $U^{d}/U$ increased toward 1.

## 4. Discussion

Figure 13 shows the difference in dilatancy angle evolution between the mudstone and the sandstone, where the mudstone demonstrates negative ψ–$\sigma_{3}$ relationships but positive ones for the sandstone. Theoretically, the dilatancy angle ψ describes the material’s volume dilation property under shearing loads. Within the positive range, the smaller the dilatancy angle, the lower the volume dilation rate. When the dilatancy angle approaches zero, volumetric plastic deformation vanishes, and the deformation gradually transforms to pure shear mode. Moreover, the volume change characteristics of rocks during the post-peak loading phase are relevant to the failure pattern. Thus, in this part, we aim to link the evolution behaviors of the dilatancy angle with specimens’ post-peak deformation and failure behaviors.

Previous research has found that the change in dilatancy angle can be characterized by the evolution of plastic shear strain [52]. However, the value of the plastic shear strain varies significantly among specimens. For the sake of simplicity, we normalized the plastic shear strain by Equation (13).

(13) $$\gamma_{n}=\gamma^{p}/\gamma_{\max}^{p}$$

where $\gamma_{\max}^{p}$ is the maximum plastic shear strain of a specimen during loading.

**Figure 16:**
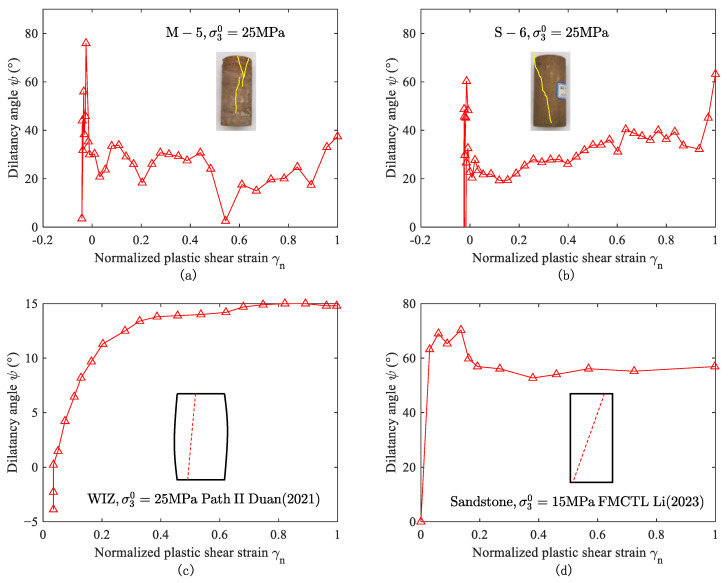
Dilatancy angle versus normalized plastic shear strain in triaxial unloading tests conducted on (**a**) the red-bed mudstone, (**b**) the red-bed sandstone, (**c**) a weak interlayered zone (WIZ) material from Duan et al. [54], and (**d**) a coarse-grained yellow sandstone from Li et al. [55].

Then, we observed how the dilatancy angle evolved with the normalized plastic shear strain. As shown in Figure 16a, specimen m-5 experiences a dilatancy angle decrease as $\gamma_{n}$ gradually increases during unloading. Correspondingly, the specimen fails in a composite shear pattern. As can be seen, three independent cracks first emerge in the specimen and gradually propagate, leading to sustained volume dilation. As the cracks develop to some extent, a localized shear sliding zone forms and the failure pattern of the specimen finally converts to shear failure. This failure pattern conversion process is companied by the change in the deformation behavior of the specimen from crack expansion to closed surface slide, where irreversible volume dilatancy subsides and shear deformation dominates. Accordingly, the dilatancy angle ψ decreases gradually. At the end of loading, the failure-induced volume expansion causes the dilatancy angle to slightly rises again.

Taking specimen s-6 as an example, Figure 16b depicts how the dilatancy angle of the red-bed sandstone changes during triaxial loading and unloading. Contrary to m-5, the dilatancy angle of specimen s-6 continuously grows with the increase in $\gamma_{n}$. After the volume dilatancy point, a localized shear damage zone gradually forms; during post-peak loading, the localized damage zone evolves into a shear slide surface. Since the red-bed sandstone is a medium-grain material, shear dilatancy happens as the specimen slides along the shear surface [53]. As the confining pressure continuously decreases in this process, the constraints on the shear dilatancy deformation continuously weaken; thus, the dilatancy angle increases. When $\gamma_{n}$ reaches 0.6, a stable shear slide surface forms, the rock blocks slide along the surface steadily, and the dilatancy angle correspondingly gets stable. At last, the dilatancy angle climbs up due to the specimen’s final failure.

In addition, we investigated some published research about the evolution of the dilatancy angle under triaxial unloading conditions and reproduced the results in Figure 16c,d. As shown in Figure 16c, Duan et al. [54] conducted a series of triaxial unloading tests on a weak interlayered zone (WIZ) material, and the axial stress was continuously increased during confinement unloading. Throughout the loading process, the specimen exhibits significant volume dilatancy and a steep shear failure surface finally forms. As a result, the dilatancy angle monotonically grows with an attenuative rate and almost keeps invariable at the end of loading. Li et al. [55] executed a group of triaxial unloading tests on a coarse-grained yellow sandstone collected from Sichuan Province, China. Similar to the studied red-bed sandstone, the specimen fails in a shear slide pattern. Meanwhile, the dilatancy angle first decreases when $\gamma_{n}$ increases from 0.15 to 0.4 and then slightly increases until specimen failure, as shown in Figure 16d. However, the dilatancy angle of the course-grained sandstone from Li et al. [55] is much larger than that of the studied sandstone during post-peak loading, which means granular rocks with larger grain sizes may exhibit more significant dilatant deformation [56].

**Figure 17:**
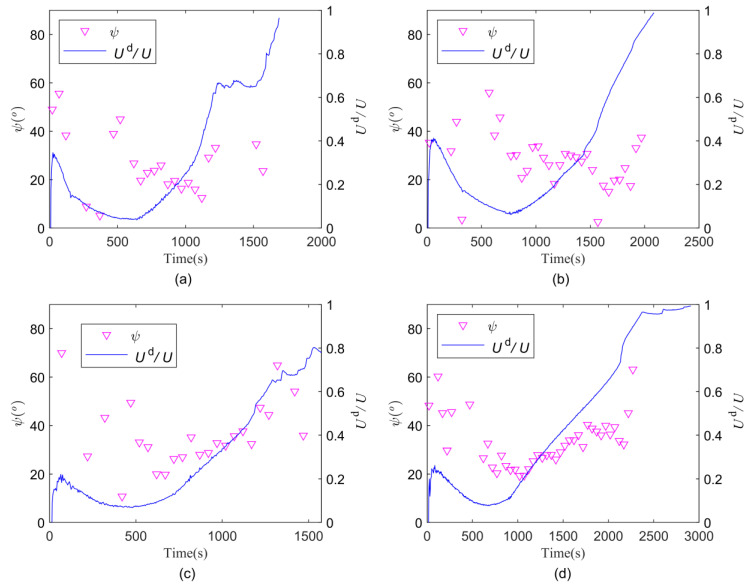
Evolutions of dilatancy angle and dissipated energy ratio with time of (**a**) m-1, (**b**) m-5, (**c**) s-1, and (**d**) s-6.

On the one hand, the previous analyses proved that the evolution of the dilatancy angle is related to the deformation and failure process of the specimen. On the other hand, the deformation and failure of the rocks are driven by energy dissipation. Thus, we further analyzed the evolution processes of the dilatancy angle and dissipated energy. As shown in Figure 17a,b, the dilatancy angle of the mudstone continuously decreases with the elapsed time, whereas the dissipated energy ratio first reduces and then grows towards 1. For the mudstone, the evolution of the dilatancy angle seems irrelevant to the dissipated energy ratio. As depicted by Figure 17c,d, however, the dilatancy angle and dissipated energy ratio of the sandstone first decrease and then increase gradually. The inflection points of the dilatancy angle slightly lagged behind those of the dissipated energy ratio regardless of different confining pressures. Moreover, the dilatancy angle of the sandstone increases steadily as the dissipated energy ratio rises linearly, and fluctuation in the dilatancy angle appears simultaneously when the dissipated energy ratio changes nonlinearly. Thus, we believe that relevance should exist between the evolutions of the dilatancy angle and dissipated energy, and more comprehensive studies are needed to make it clear whether the correlation between the dilatancy angle evolution and the energy dissipation process found in our research is universal or just a coincidence.

## 5. Conclusions

In terms of volume dilation and energy conversion, we investigated the mechanical responses of two red-bed rocks under unloading conditions through laboratory tests in this study, and the main conclusions can be drawn.

The mudstone and sandstone experienced significant volume dilatancy under unloading. The dilatancy stress increased linearly with the initial confining pressure. However, the ratio of dilatancy stress to the peak stress kept stable within a range of 0.8~0.9, regardless of the varying confinement.

Essentially, confinement unloading reduced the restriction on the expansion of microcracks and resulted in strong volume dilatancy. As the confining pressure was continuously unloaded, the lateral plastic strain accumulated acceleratively, and the dilatancy angle of rocks changed linearly. However, the evolution of dilatancy angle exhibited significant differences between mudstone and sandstone. Under confinement unloading conditions, the dilatancy angle of the mudstone decreases linearly with the confining pressure, whereas the sandstone behaves oppositely.

The energy conversion process of the rocks under triaxial unloading conditions can be divided into three phases, where the dissipated energy first kept relatively stable, then grew steadily, and climbed up as the post-peak stress dropped. Finally, almost all the input energy was consumed by rock deformation and fracturing. For both rocks, more energy was consumed under higher confining pressure, and the hard sandstone took more energy during deformation than the soft mudstone.

Since the change in dilatancy angle is related to the irreversible crack propagation in the rock, the correlation between the varying dilatancy angle and the rock failure process was discussed. Sandstone failed in a splitting–shearing mode, but mudstone failed with a pure shear surface. Two failure modes correspond to different evolution processes of cracks, thus resulting in totally opposite trends of dilatancy angle variation. Moreover, for the first time, the links between the energy dissipation and evolution of dilatancy angle during confinement unloading were observed, but the reason needs further investigation.

The results can help better understand the mechanical behaviors of the red-bed rock under unloading conditions and provide scientific instructions for engineering practice. Also, some interesting findings in this study may provide a new perspective to comprehend the volume dilatancy and energy dissipation behaviors of red-bed rock.

## Figures and Tables

**Table 1:** Basic physical parameters of the rocks, in the bracket is the average value.

Rock Type	Natural Density (kg/m^3^)	Natural Water Content (%)	Average Porosity (%)	P-Wave Velocity (m/s)
Mudstone	2610~2640 (2630)	1.87~2.35 (2.14)	1.67	1376~3406 (2663)
Sandstone	2660~2690 (2680)	2.78~3.12 (2.53)	6.03	3774~4739 (4214)

**Table 2:** Mass proportions of rocks’ mineral components based on XRD and XRF analyses.

Rock Type	Mineral Name (%)								
	Quartz	Feldspar	Calcite	Dolomite	Muscovite	Chlorite	Hematite	Kaolinite	Others
Mudstone	33.3	11.1	12.7	10.5	4.7	7.3	4.4	10.8	5.2
Sandstone	34.4	20.8	16.5	15.9	1.5	1.5	1.7	-	7.7

**Table 3:** Mechanical parameters of the mudstone and sandstone under unloading conditions in TUCP tests.

Rock Type	Sample Name	$\sigma_{3}^{0}$	*E*	*ν*	$\sigma_{3}^{p}$	$\sigma_{1}^{d}$	$\sigma_{1}^{p}$	$\varepsilon_{v}^{p}$	*c*	*φ*	*c*_0_	*φ*_0_
		MPa	GPa		MPa	MPa	MPa		MPa	°	MPa	°
Mudstone	m-1	10	6.14	0.31	4.80	43.38	49.81	0.0021	6.55	40.57	11.89	38.22
	m-2	10	6.01	0.29	5.25	43.68	48.27	0.004				
	m-3	20	6.96	0.30	14.82	66.06	73.48	0.0035				
	m-4	20	7.79	0.31	14.80	66.11	75.35	0.0029				
	m-5	25	11.81	0.30	17.49	72.62	99.12	0.0017				
	m-6	25	12.49	0.28	17.27	84.08	101.76	0.0029				
Sandstone	s-1	10	20.78	0.20	6.65	90.60	106.55	0.0009	26.64	33.49	19.26	47.03
	s-2	10	36.59	0.20	6.89	99.10	123.78	0.0002				
	s-3	20	25.91	0.21	20.00	118.47	144.00	0.0015				
	s-4	20	23.75	0.20	14.98	115.63	139.29	0.0004				
	s-5	25	22.44	0.21	19.06	123.83	147.91	0.0009				
	s-6	25	21.32	0.21	19.26	124.39	147.01	0.0013				

Note: *c*_0_ and *φ*_0_ are the cohesion strength and the friction angle determined from CTC tests.

## Data Availability

The experimental data adopted in this research are available from the corresponding author on reasonable requests.
